# Multi-purpose reservoir operation oncomitant with estimating hydropower potential using multifarious hydrological models

**DOI:** 10.1016/j.heliyon.2023.e23821

**Published:** 2023-12-18

**Authors:** Yordanos Mekuriaw Meskr, Abdella Kemal Mohammed, Abebe Temesgen Ayalew, Tarun Kumar Lohani

**Affiliations:** Water Technology Institute, Arba Minch University, P.O. Box. 21, Arba Minch, Ethiopia

**Keywords:** HBV-Light, HEC-ResSim, Hydropower, Rib watershed, Reservoir operation

## Abstract

The research aims at determining the optimal release rule to increase the capacity of Rib reservoir. The reservoir inflow using HBV-light hydrological model embracing optimal reservoir operation through HEC-ResSim model were used to prepare an optimum operational plan. The potential of the river for hydropower generation prioritise the demand at a specified level regarding storage capacity (m^3^), level of reservoir (m), and the relation between inflow and outflow of the reservoir. From the model performance features, the coefficient of correlation (R2) and Nash Sutcliffe Efficiency (NSE) were determined to be, respectively, 0.77 and 0.73 for calibration and 0.72 and 0.70 for validation. The Sobol approach was used for detailed sensitivity analysis of DROP model parameters based on the performance of C2M on outflows and volumes. The results suggest that the threshold coefficient characterizing the demand-controlled release level is the most significant parameter. According to the simulation's findings, the reservoir's average regulated release is calculated to be 22.86 m^3^/s, and its average monthly hydropower output is 6.73 MW. Average annual hydropower energy was estimated as 58.955 GW h/year and mean annual inflow of reservoir volume of water to be 223.54 Mm^3^. This volume of water is adequate to accommodate total annual irrigation demand, environmental obligation, and other respective requirements in the downstream. The demand for hydropower and irrigation and supply from reservoir capacity can be counterbalanced from the simulated result without any hindrance.

## 1 Introduction

Water resources management is the backbone for the economic growth of a developing country [[1], [2], [3], [4], [5]]. The day-to-day activities of all human beings have been deeply connected with water which can be compensated from reservoirs built on streams or rivers to fulfil during the rainy season and meet various demands during dry seasons [[6], [7], [8]]. Ethiopia utilizes its irrigation and hydropower potential to boom the economic development of the country (Reda et al., 2015; [[9], [10], [11], [12], [13]].

Reservoirs that have both hydropower and irrigation functions serve multiple purposes. They act as buffers for fluctuations in natural stream flow, helping to control the water supply and release it as needed for different purposes. During dry periods, such reservoirs can provide a reliable water supply for irrigation, ensuring that agricultural needs are met even when there's a scarcity of natural water sources. Additionally, these reservoirs are operated in a way that allows water to be stored, creating hydraulic head, which can be released later to generate hydroelectricity. This dual functionality makes them valuable in terms of both water resource management and renewable energy production [[14], [15], [16], [17]]. Conflicting water use among different users is indeed a significant challenge in multi-purpose reservoir operation. Reservoirs often serve various purposes such as irrigation, hydropower generation, municipal water supply, flood control, and ecological maintenance [[18], [19], [20], [21]]. Each user has different needs and objectives, resulting in conflicting demands for water. To address this challenge, optimizing the operation of multi-purpose reservoirs is crucial. This involves finding a balance between the competing demands to achieve the best possible outcome for all users while considering conflicting objectives.

One of the key challenges in water requirement for agriculture and hydropower is the mismatch in demand seasonality [1,7,22,23]. This conflict is exacerbated during dry seasons when water availability is low, particularly along the Rib water basins. This study uses the HEC-ResSim model to simulate the reservoir and apply a reservoir operating rule curve in order to calculate the hydropower potential. The generated power can be used to electrify rural communities and improve their living standards, without compromising the irrigation water demand. Currently, most rural villages in Ethiopia lack access to electricity and rely on traditional energy sources such as biomass energy and petroleum products. This hinders the development of rural livelihoods and their ability to escape poverty. Additionally, many reservoirs lack pre-determined and updated operation rule curves, which leads to ineffective operation and failure to meet desired objectives. So this study aims to maximize the benefit of the Rib irrigation dam by including Hydropower, whereas the Rib irrigation Dam proposed to store and release water for only irrigation purposes without producing hydroelectric Power. Therefore, the turbine or the powerhouse can be located on the downstream side of the dam at the toe and can generate hydroelectric power using the irrigation outlet as a penstock without affecting the current irrigation requirements.

## 2 Materials and methods

### 2.1 Area of study

**Fig. 1:**
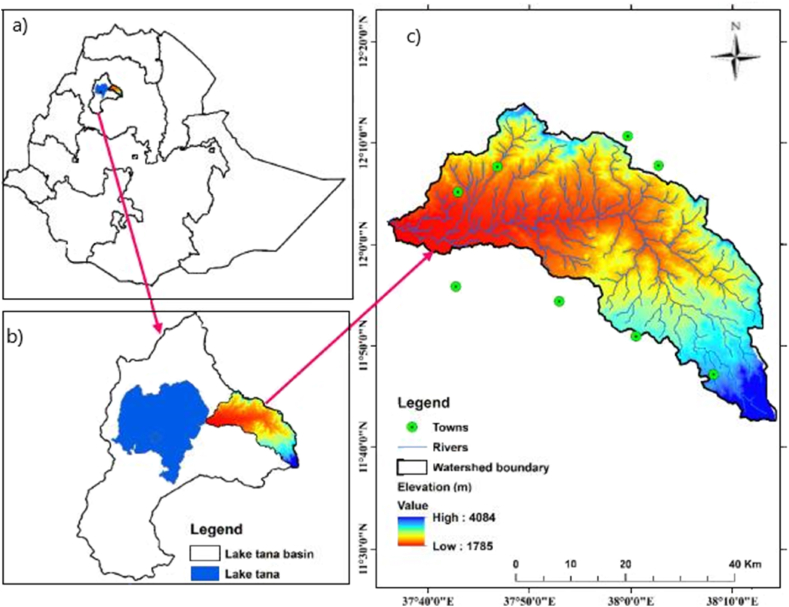
Study area: a) Map of Ethiopia; b) Tana Lake Basin; c) Watershed.

The Rib River watershed is situated in the South Gondar zone of the Farta, Ebinat, and Debre Tabor woredas in the Amhara National Regional State. It is located 60 km from Bahir Dar and 625 km northwest of Addis Ababa. The area's geographic coordinates are 12° 35′ N, 41° 25′ E, 13° 54′ N, and 35° 55′ E. The area of study represented in Fig. 1.

### 2.2 LULC, climate and soil type categorization

According to the maximum likelihood classification of 2019 land sat satellite image in Upper Rib Watershed (Reservoir site area), the proportion of cultivated land for cultivation 52.94 % bush and shrub lands 31.306 %, grazing land 10.297 %, woody savanna land 2.069, forest cover 1.57 %, urban and settlement Area 1.406 % and water body 0.412 % [24]. The major Soil type distribution of the upper Rib catchment is described in Table 1.

**Table 1:** Soil distribution in upper rib river catchment.

S.No.	Soil type	Area (ha)	% of the watershed area
1	Chromicluvisols	38630.8	56.4
2	Eutric Fluvisols	1826.2	2.7
3	Eutric Leptosols	28,043	40.9

**Table 2:** Summary of the DROP model parameter default values and feasible ranges for the sensitivity analysis [25].

Parameter	Default value	Minimum Value	Maximum value	Distribution
α	0.85	0.6	0.95	uniform
dmax (km)	100	1	250	uniform
Mstart (Hydropower: other)	4; 5	1	12	Discrete uniform
M	0.5	0	1	Uniform
Cthrehold	0.5	0.001	20	Logarithmic
b	2	0.5	5	Uniform

### 2.3 Materials used

The study utilized several materials, models, and software tools for various purposes (see Table 2). Here is a breakdown of each component and its specific role:

**Google Earth Pro**: used to gain a general overview of the study area before conducting the actual observation of the site area.

**ArcGIS**: utilized to process DEM data for the extraction and delineation of the catchment area. ArcGIS has versatile capabilities for handling spatial data and performing various analyses.

**FAO ETo Calculator**: used to calculate potential evaporation, which was an input for the hydrological model HBV-Light.

HBV-Light Hydrological Model: used to simulate runoff based on inputs, including potential evaporation estimates obtained from the FAO ETo Calculator and other relevant data.

**HEC-ResSim Model**: utilized the simulated runoff as input to simulate reservoir operation. This model enables the assessment and optimization of reservoir operations.

**HEC-DSSVue**: employed for data entry, storage, and analysis of outputs generated by the HEC-ResSim model. It enhances the visualization and interpretation of simulation results.

**Excel Spreadsheet**: used for further analysis of the outputs produced by the HEC-ResSim model. Excel provides a versatile platform for data manipulation, calculations, and visualization.

#### 2.3.1 Methodology

**Fig. 2:**
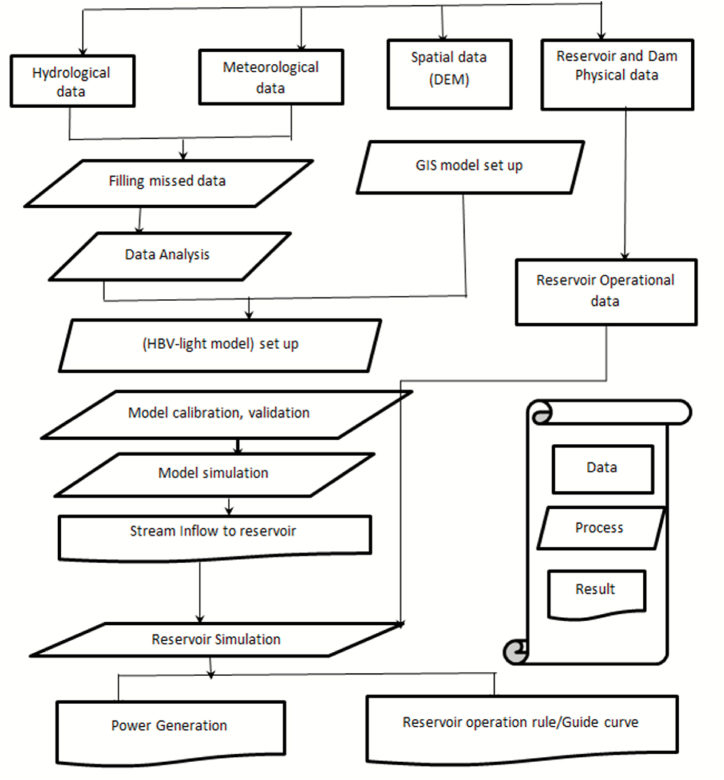
General framework of the study.

In Fig. 2, the research's general methodological procedure is displayed.

### 2.4 Hydro meteorological data

To complete the documentation gaps, the Addiszemen station's hydrological data was gathered between 1988 and 2015. The hydrological HBV-light model assists in simulating daily discharge at the dam site and the operation of the Rib reservoir since reservoir inflow data is the most important input data for the HEC-ResSim reservoir simulation model. The hydrological model HBV-light was employed to determine the stream flow into the Rib reservoir.

### 2.5 Homogeneity test

**Fig. 3:**
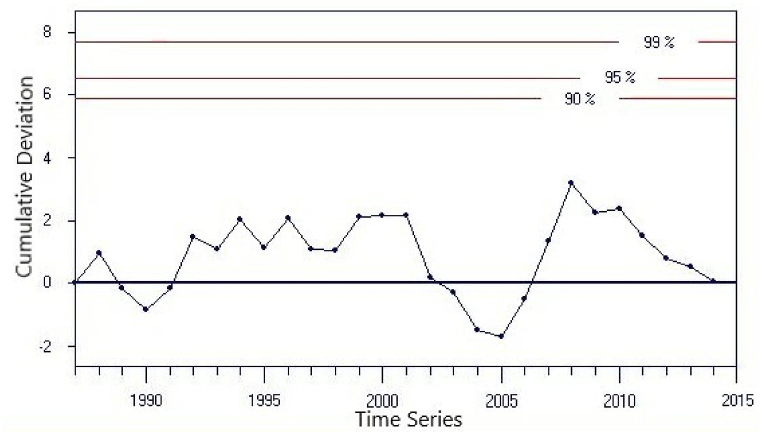
Cumulative deviations of total annual Flow and probability of rejecting Homogeneity of annual flow using Rainbow test at Addis Zemen gauging station.

The homogeneity of historical stream flow data sets is analysed and evaluated by a piece of software called Rainbow. Rainbow uses frequency analysis to examine weather or hydrologic records and to make sure that the observations in the data set come from the same population (Davy et al., 2020; [26]. The homogeneity of the annual flow for the gauged station at Rib River using Rainbow is presented Fig. 3.

**Fig. 4:**
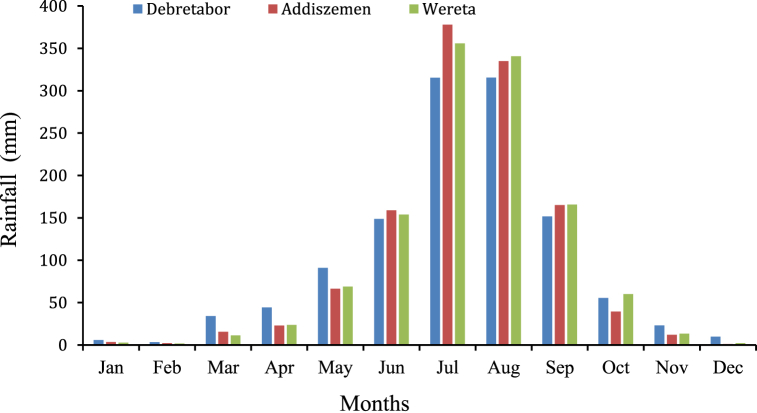
Mean monthly rainfall (mm).

Meteorological data required in this study are the daily rainfall data, daily mean temperature, daily discharge, monthly mean Temperature and monthly mean evaporation data of the selected metrological stations are inputs for HBV-light hydrological model and the detail mean monthly base of rainfall data is mentioned in Fig. 4. This model simulates runoff at the dam site to estimate inflows into the Rib reservoir; this estimated inflow is then used as an input for the HEC-ResSim reservoir simulation model to calculate the inflow and outflow of the reservoir, the reservoir's operational guidelines, and to calculate the reservoir's hydroelectric potential.

### 2.6 Homogeneity of meteorological stations

**Fig. 5:**
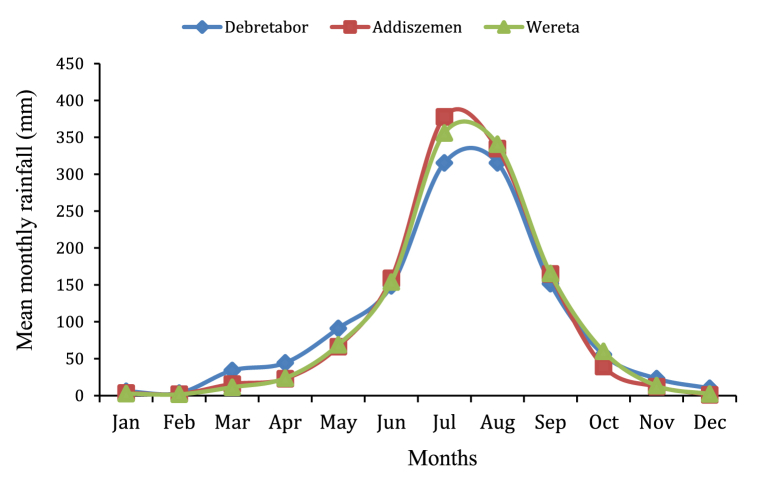
Rainfall homogeneity of the selected Station.

The monthly rainfall records were carried out by checking homogeneity of selected group stations which are non-dimensional [27,28] (Fig. 5). This model was used to simulate runoff at dam site to estimate inflows into the Rib reservoir, and this estimated inflow is an input for HEC-ResSim reservoir simulation model, determine inflow and outflow of reservoir, reservoir operation rule, and to estimate hydropower potential of the reservoir (Equation (1)).

(1) $$Pnon=\left(\frac{Pi}{Pave}\right)*100\%$$

where: P *non* = Value of precipitation for non-dimensional for the month i.

*Pi* = monthly precipitation over years averaged for the station i.

*Pave* = yearly precipitation for the over years average of the station.

**Fig. 6:**
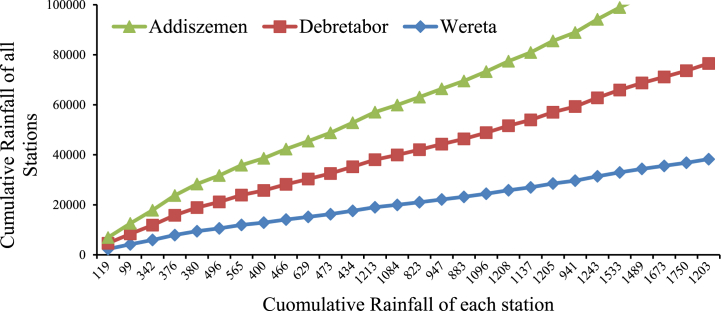
Consistency test (Double Mass Curve) for selected Meteorological Stations.

**Test for consistency of rainfall record by using Double mass curve**: A graph (Fig. 6) comparing the cumulative catch at the interest rain gauges versus the cumulative catches of one or more gauges in places that have seen comparable hydro-meteorological occurrences and are recognized to be consistent is called a double-mass curve (Equation (2)) [29,30].

(2) $$Px=\frac{Px^{\prime }\;*\tan\;\alpha^{\prime }}{\tan\;\alpha }$$

where**:***Px* - Adjusted Rainfall of station, *Px*’ - Observed Rainfall at station x, tan α′ - The slope of the adjusted mass curve, tan α – The slope of the Original mass curve.

### 2.7 HBV-light modeling and analysis

**Fig. 7:**
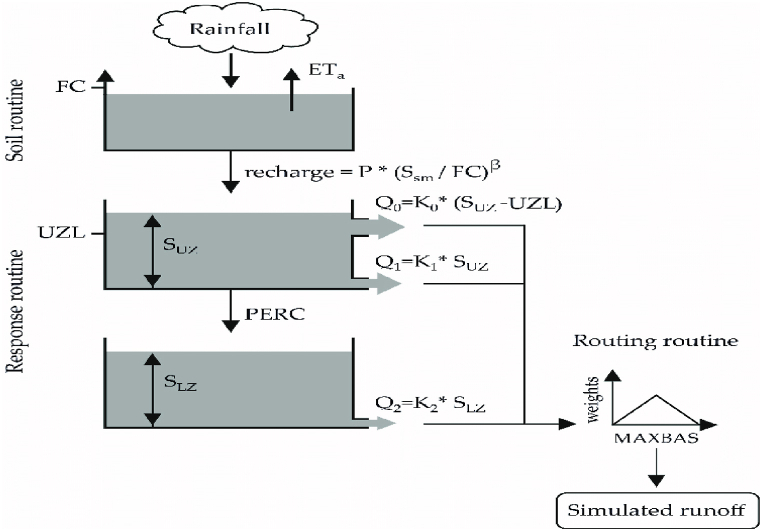
Structure of the HBV-light model for this study.

In this study HBV-Light version 4.0.0.**17** was used instead of the old HBV version because its simplicity in research and education with minimal input data [31]. The soil moisture routine in HBV-Light is primarily governed by three main parameters: BETA, LP, and FC [32]. Finally, the runoff generation routine in HBV-Light acts as a response function, converting excess water from the soil moisture zone to runoff (Fig. 7).

### 2.8 Calibration and validation of HBV-light model

When calibrating a model, input parameters are changed, and the output simulation runoff is compared to observed runoff values until the objective function is met [33]; Davy et al., 2020). The calibration was conducted by trial and error where model parameters are manually or automatic calibration. So, in this study manual calibration method was used for a period 1990–2001 for calibration and 2002–2015 for validation with warm-up period (1988–1989). 28 years of flow data were used from (1988–2015) the Validation test of model performance with calibrated parameters for an independent period and in this study fourteen years was used for validation from (2002–2015) and calibration period from (1988–2001) was used.

### 2.9 Model performance

A model is calibrated by altering the input parameters and then comparing the simulation's output runoff values to actual runoff measurements until the goal function is achieved.

**Nash-Sutcliffe efficiency (ENS):** It helps to judge the fit concerning the outcome of the model and actual measured hydrograph shapes [20]. The effectiveness of the model is determined by ENS. ENS can have a value between one and -, with one being the optimal value. Values between 0.80 and 0.90 indicate the model performs admirably, while values between 0.90 and 1 indicate the model performs extraordinarily well. (Girmay et al., 2021).

**Coefficient of determination** (R^2^): R^2^ reflects the model approach to recreate the observed value through a given time period and for a given time step. R^2^ values vary from 1.0 (best) to 0.0 [26].

**Percentage of bias (PBIAS):** The predisposition of anticipated threshold higher/smaller than measured value is assessed by percent bias (PBIAS) (Tufa et al., 2021). The absolute value of PBIAS should be as low as possible for a well-performing model.

**The ratio of root mean square error to observation standard deviation (RSR)**: It serves as an error index indicator [29]. RSR has a value between zero and one, with the lower value, closer to zero, suggesting better model representation and one indicating poor model performance (Kwakye et al., 2022).

### 2.10 Reservoir simulation model (HEC-ResSim) description and data preparation

The planning and management of the water resources system include reservoir operation [34]. Three distinct sets of functions, referred to as modules, are provided by HEC-ResSim and give users access to various categories of data within a watershed. Each module serves a distinct role, and there is a corresponding set of tool available through menus, toolbars, and schematics. The HEC-ResSim reservoir simulation model is unusual among reservoir simulation models with an aim to replicate the decision-making process that human reservoir operators must employ to plan releases. HEC-ResSim provides three distinct sets of functions known as modules that allow access to various sorts of data within a watershed. Watershed Setup, Reservoir Network, and Simulation are the components involved. Each module has a distinct role, as indicated below.

**Watershed Setup Module:** The watershed setup module includes several display applications and a framework for generating watersheds [35]. This module allows users to incorporate streams, reservoirs, gauge locations, time-series locations, and hydrologic and hydraulic data specific to a particular area. The watershed setup module creates a thorough framework for analysing and comprehending the hydrological properties of a watershed by specifying these specifics. The Watershed Setup module's goal is to provide a consistent framework for watershed formation and specification across various modeling programs. HEC-ResSim presently uses this module [18]. A watershed is related to a geographic region that may be designed with numerous models and area coverage. A watershed may encompass a certain area's streams, projects (reservoirs, levees), gauge locations, impact regions, time-series locations, and hydrologic and hydraulic data. When all those features are combined, they constitute a watershed framework. Items that define the physical layout of a watershed are constructed in the Watershed Setup module. After creating a new watershed, one can import maps from other sources, set the units of measurement for viewing the watershed, add layers with extra information about the watershed, create a common stream alignment, and customize components.

**Reservoir Network Module:** In the reservoir network module, river schematization, description of the physical and operational elements of the reservoirs can be built, and alternatives are developed and also add Routing reaches, Junctions possibly other network elements [21]. The physical and operational data for each network defined after the reservoir connectivity network schematic is accomplished. The Reservoir Network module's objective is to separate reservoir model creation from output analysis [19,20]. The Reservoir Network module creates a river schematic to define the physical and operational features of the reservoir model and produce the alternatives that will be analysed. Using the configurations generated in the Watershed Setup module as a template, the base of a reservoir network is then needed. To complete the network schematic's connection, routing reaches and perhaps other network elements are included. Physical and operational data for each network piece are defined after the design is finalized. Alternatives are also constructed that specify the reservoir network, operation set(s), beginning circumstances, and DSS pathname assignment (time series).

**Simulation Module:** Simulation module provides the users to configure and create a simulation result through existing reservoir network and user-defined alternatives with specified time intervals of 1 day [36]. The goal of HEC-ResSim was to ascertain the reservoir operation released over a specified time period for a particular multi-purpose reservoir with known stream flow at the reservoir's input point and various control points throughout the system. The purpose of the Simulation module is to isolate output analysis from the model development process [21]. Once the reservoir model is complete and the alternatives have been defined, the Simulation module is used to configure the simulation. The computations are performed and results are viewed within the Simulation module. When creating a simulation then following must specified; a simulation time window, a computation interval, and the alternatives to be analysed. Then, HEC-ResSim creates a directory structure within the Dss folder of the watershed that represents the “simulation”. Within this “simulation” tree will be a copy of the watershed, including only those files needed by the selected alternatives. Also created in the simulation, is a DSS file called simulation. dss, which will ultimately contain all the DSS records that represent the input and output for the selected alternatives.

### 2.11 HEC-ResSim input data

**Reservoir-Storage-Elevation-Area curves:** Reservoir-storage-elevation curves, also known as stage-storage-area curves, provide information about the relationship between the elevation (height) of water in a reservoir, the storage volume of water, and the surface area of the reservoir at different elevations [37]. These curves help to understand the properties and characteristics of the reservoir. On the other hand, the length and elevation of the crest of the dam that creates the reservoir are used to characterize it. The crest elevation refers to the highest point of the dam, which determines the maximum water elevation that the reservoir can reach. The length of the dam represents the distance along the top of the dam from one end to the other. Both the elevation-storage-area curves and the dam characteristics are important in understanding and managing reservoir systems.

**Reservoir evaporation:** Reservoir evaporation Contributes a great share of the total loss from a reservoir the evaporation data series was determined using the Penman-Monteith method on long-term mean monthly [38]. For this study, evaporation of Rib reservoir was determined based on the record of Debretabor weather station.

**Time-series flow data:** Daily flow data was gathered from MoWIE at the Addiszemen gauging station, but the inflow into the Rib Reservoir was approximated at the Rib dam site using the HBV-light model. The simulation model HEC-ResSim received this simulated daily discharge data as input.

**Operational parameters**: Operational parameters of the Reservoir are Zone, Rule, and Guide curves. Zones are based on specific reservoir elevations based on the total storage level, depending on this flood control zone, conservation zone, and dead storage zone have been set at 1945.5, 1940, and 1892 a.m.s.1 respectively. For each mode of operation, the rules are ordered by first priority demands. The conservation zone is the preferred zone for reservoir operations. In HEC-ResSim, the power plant parameters are used to define the hydroelectric power generated by the turbines. The total installed capacity of the Rib hydropower plant is 21 MW, station Use 0, total head loss assume 3.5 m, overall efficiency 85 % and tail water elevation used is 1875 m a. s.1.

### 2.12 Sensitivity analysis implementation

A sensitivity analysis comprising of six parameters was conducted on the performance [20] and bounded version, called C_2M_ [15] on outflows using the Sobol method. In fact, the NSE values in some reservoirs were highly negative for some simulations, and thus this metric was not suitable for a variance-based sensitivity analysis method like Sobol's. C_2M_ is used instead as it is a normalized version of NSE that varies between −1 and 1 and where all negative values are bounded between 0 and −1. Parameter default values, bounds and distributions are listed in (Table 2). The parameter distributions were all considered uniform except for relative capacity, for which the distribution is logarithmic to align with the observed pattern on the modeled reservoirs.

## 3 Results and discussions

### 3.1 HBV-light hydrologic model results

**Fig. 8:**
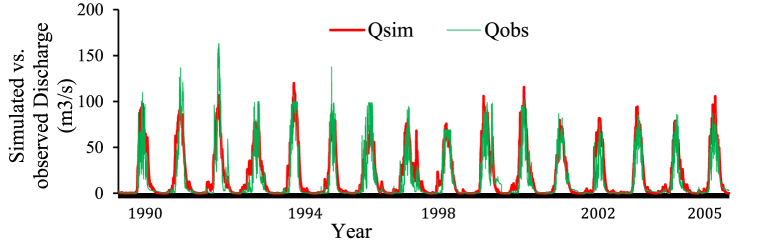
Flow in (m^3^/s) that was observed and simulated throughout the calibration period.

The 28-year observed data period (1988–2015) is used to calibrate and validate the HBV-light model, and the best-fit parameter values are chosen. Daily data from 1988 to 2005 were used for calibration, daily data from 2006 to 2015 were used for validation, and daily data from 1988 to 1989 were used for warming up the model (warm-up period). The results of the calibration show that the HBV-light model's performance in simulating the Rib watershed is satisfactory when objective functions like NSE and R_eff_ are greater than 0.60, but in this study, R_eff_ (R^2^) = 0.77 and NSE = 0.73 were used for the entire calibration period. As a result, NSE and R^2^ are within acceptable ranges (Fig. 8). The parameter values of HBV light model is presented in Table 3.

**Table 3:** Parameters and values for the HBV-light model after calibration.

parameter	Valid Rang	Optimized value		
		Vege. zone-1	Vege. zone-3	Vege. zone-3
FC	(0,inf)	200	200	200
LP	(0,1)	0.68	0.68	0.68
BETA	(0,inf)	3	3	3
PERC	(0,inf)	100	100	100
UZL	(0,inf)	10	10	10
K0	(0,1)	0.08	0.08	0.08
K1	(0,1)	0.05	0.05	0.05
K2	(0,1)	0.08	0.08	0.08
MAXBAS	(1,1000)	2	2	2

### 3.2 Flow into rib reservoir

**Fig. 9:**
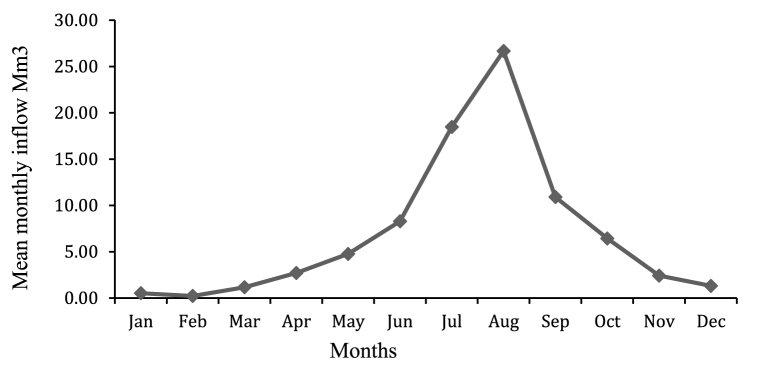
Rib Reservoir Mean monthly Inflow Values (Mm^3^***)***.

HBV-light rainfall-runoff model simulates runoff at dam site by using daily precipitation daily discharge, mean temperature, and monthly potential evaporation, and this simulated flow was important input for HEC-ResSim model for Reservoir simulation purpose. Mean monthly inflow volume of Rib reservoir as shown in Fig. 9 vary from month to month, but variation are large, maximum monthly inflow is 71.47 Mm^3^ in August and minimum monthly inflow 0.54 Mm^3^ in February, and annual inflow is 223.54 Mm.^3^

### 3.3 HEC-ResSim simulation model results and discussions

**Fig. 10:**
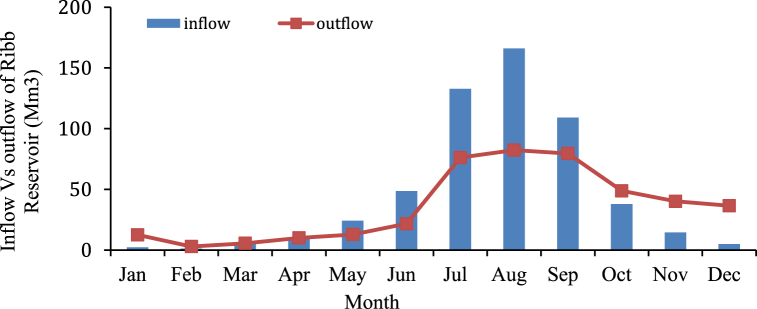
Inflow-outflow hydrograph of rib reservoir.

**Inflow and outflow using HEC-ResSim:** The total outflow from Reservoir through irrigation outlet (209.59 Mm^3^) and mean annual inflow (223.54 Mm^3^). Therefore, total outflow are less than mean annual inflow this indicates that operating the reservoir throughout the year does not have an impact on both irrigation and environmental demands including Hydropower release. The storage capacity, which was previously estimated, was 234 Mm^3^ during Rib dam design. Generally, hydropower can be generated throughout the year using the irrigation outlet as a penstock when hydropower plant is annexed at existing irrigation dam. Whereas while, Simulation results indicated that the maximum controlled or regulated flow release capacity was 40 m^3^/s and average release from the reservoir is 22.86 m^3^/s (Fig. 10).

### 3.4 Reservoir operation guide curve

**Fig. 11:**
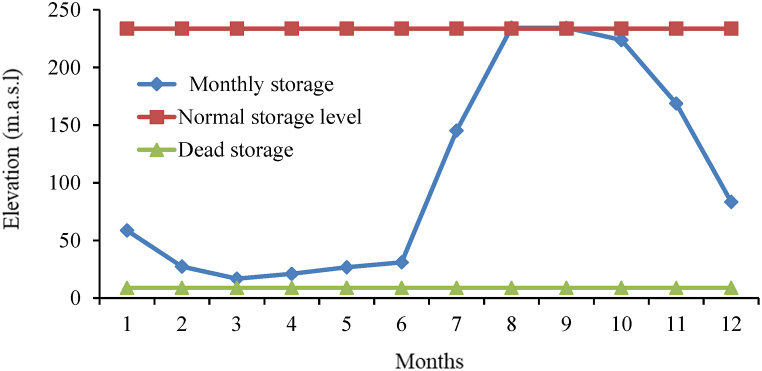
Monthly Average storage Rule curve.

From the Reservoir simulation HEC-ResSim model results in mean monthly storage, normal water level of reservoir, and operating reservoir storages, and the corresponding elevations dead level, conservation level, and average elevation over the entire period of analysis were obtained from HEC-ResSim simulation model results of storage-elevation curve (Fig. 11). By using the Microsoft Excel application to interpolating storage and elevation values of results such that mean monthly storage rule curve and operating level of the reservoir throughout simulation results (Table 4).

**Table 4:** Storage and Corresponding Elevation of the Rib reservoir.

	Storage zone				Elevation (m.a.s.l)	
Month	Mean month	Normal storage	dead storage	Operating	Conservation	Inactive
	Storage (Mm^3^)	level		level	level	level
Jan	58.79	233.7	8.945	1895.29	1940	1892
Feb	27.42	233.7	8.945	1893.54	1940	1892
Mar	16.87	233.7	8.945	1892.20	1940	1892
Apr	21.09	233.7	8.945	1892.90	1940	1892
May	26.81	233.7	8.945	1893.79	1940	1892
Jun	31.03	233.7	8.945	1900.52	1940	1892
Jul	145.31	233.7	8.945	1927.95	1940	1892
Aug	234.49	233.7	8.945	1940.11	1940	1892
Sep	234.41	233.7	8.945	1940.07	1940	1892
Oct	223.85	233.7	8.945	1938.86	1940	1892
Nov	168.87	233.7	8.945	1931.19	1940	1892
Dec	83.45	233.7	8.945	1912.19	1940	1892

**Fig. 12:**
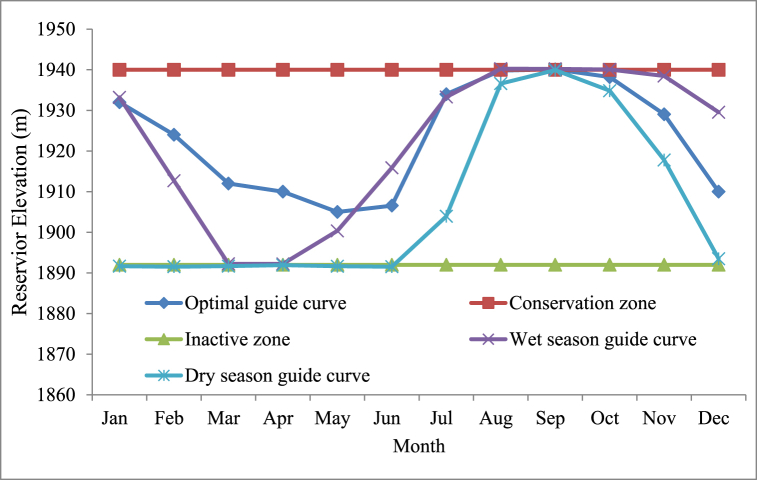
Rib Reservoir Comprehensive Guide curve for Different Hydro meteorological conditions.

**Fig. 13:**
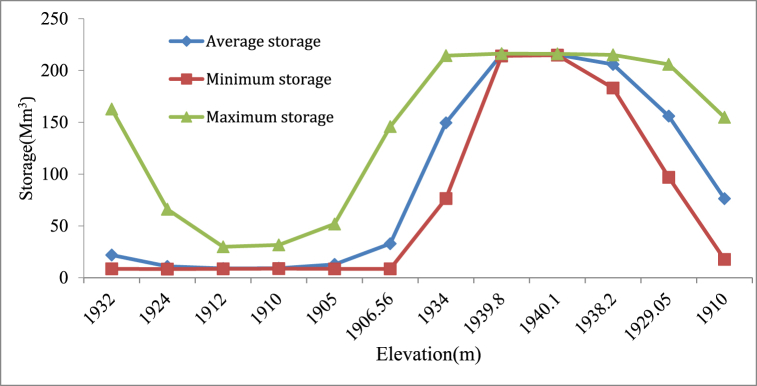
Elevation vs storage.

A dry season guide curve and a rainy season guide curve are hydrological tools for managing water resources in places with distinct wet and dry seasons [34]. Based on historical data, these curves are used to calculate the quantity of water that may be released from a dam or reservoir during different seasons. In the water resource management area, dry season guide curves and rainy season guide curves are often used to estimate and prepare for water availability throughout different seasons [37]. The dry season guidance curve (Fig. 12) is a graphical depiction of the minimum quantity of water that should be available in a reservoir during the dry season. It is estimated using historical rainfall patterns, evaporation rates, and water consumption. The dry season guidance curve's aim is to guarantee that enough water is kept in the reservoir to fulfil demand during the dry season, which has lower rainfall and greater water use. The wet season guidance curve, on the other hand, is a graphical depiction of the maximum quantity of water that should be held in a reservoir during the rainy season. The rainy season guide curve's aim is to guarantee that extra water from heavy rain events is held in the reservoir for future use while also preventing overflows and flooding. Moreover to know about the storage at different elevation (Minimum, average and maximum) storage are evaluated and described in (Fig. 13).

**Fig. 14:**
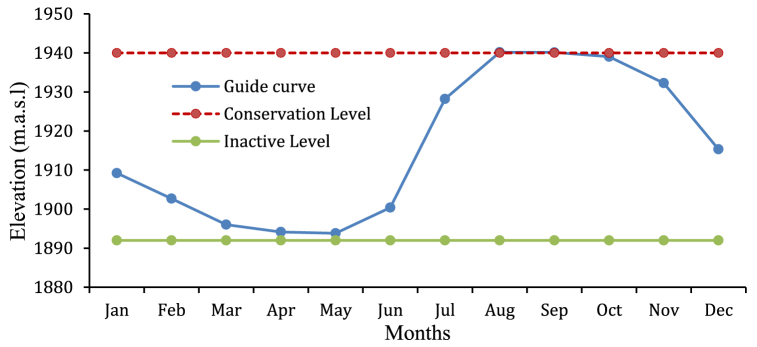
Rib Reservoir operation Guide Curve.

Rib reservoir guide curve has been developed from the water levels or storage for each month, and this guide curve can be interpreted as the most effective operation guide curve for those years of hydro-meteorological conditions. This information is intended to provide a general understanding of how and when the reservoir should have been operated in the past data, as well as some information regarding how the reservoir should be operated in the future and the actual minimum, maximum, and ideal reservoir levels at the conclusion of the operation period of the Rib reservoir for the entire years of analysis. Accordingly, the highest priority demand (Irrigation and Environmental demand) was satisfied as to the actual minimum water levels of the reservoir for all months were above the inactive (dead) storage zone level (1892 m). Observed from this values of the actual maximum water level are below the Conservation level of the reservoir for all months except for August, and September, although irrigation demands for Jun, July, August, and September of these months (rainy season) are zero, then dam operates for only hydropower generation (through irrigation outlet). The actual maximum water level in the reservoir was above the top of the conservation level but below the flood level (1945.5), as determined by the mean maximum pool level in the Rib for the months of August and September. Therefore, the extra water must be discharged through the spillway to maintain the reservoir level at its conservation level to prevent flooding (Fig. 14).

### 3.5 Hydropower potential of the Rib Reservoir

**Fig. 15:**
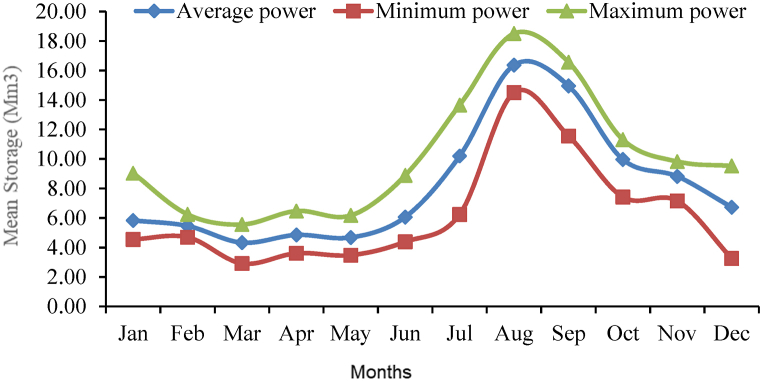
Monthly generated power production.

The daily and monthly power created from the simulation model output throughout the course of the entire year of analysis shows that there is significant daily and monthly power generation volatility caused by considerable changes in inflow and outflow [5]. During the wet season, the inflow to the reservoir is very high and the release increased to generate more power, and during dry season, inflow is less and generates less power. From the simulation result, daily maximum power generated was 17.27 MW on August 19, 2010, and daily minimum power generated was 2.84 MW on March 9, 2006 [1]. While, maximum and minimum monthly power generated was 16.36 MW in August 2010 and 4.33 MW in March 2006 respectively. However, this power generated when hydropower plant annexed at existing irrigation dam (Fig. 15).

According to the above figure, indicates that the monthly variability of the optimum power production is high, and monthly maximum power production is 16.36 MW during August 2010 whereas the smallest monthly power production is found to be 4.33 MW during March 2006, whereas monthly average hydropower production 6.73 MW at P_50_

### 3.6 Mean power duration curve

**Fig. 16:**
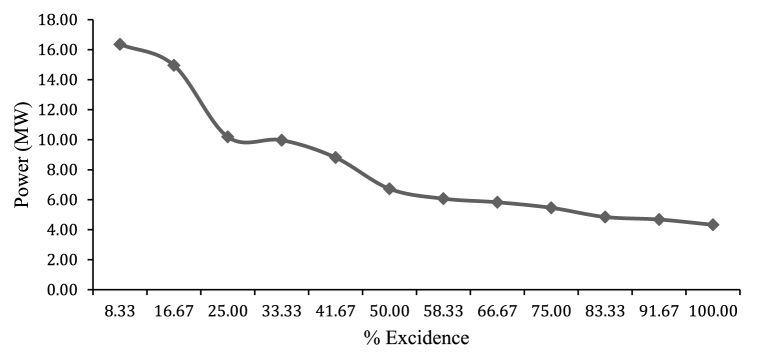
Mean Power duration Curve (MW).

Mean power duration curve defined as power production capacity at each percentage of exceedence, and shows primary and secondary power generation capacity. The resulted mean Power duration curve of the Rib reservoir gives an idea of the power generated at any instant of time in a month over a year. As shown in the figure below the minimum power produced is the firm power, which is available 100% of the time, and this minimum power is around 4.33 MW. to be available at all times, and the small power production, which available 95% of the time is 4.68 MW. It can be represented as P95. The average power production is a power, which is available 50% of the time, is 6.73 MW and it can be represented as P50, and it is called average power, etc as shown in Fig. 16. Therefore, according to power duration curve monthly average power production 6.73 MW and yearly average hydroelectric energy production is 58.95 GW h/year.

### 3.7 Sensitivity analysis of the optimal release rule under different scenarios

**Fig. 17:**
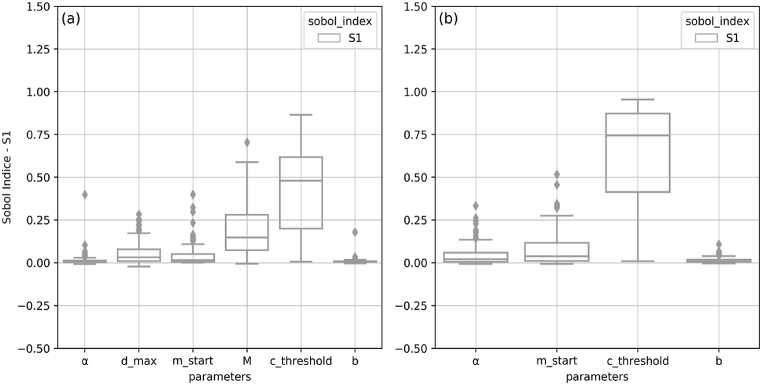
Distribution of first (S_1_)-order Sobol indices in the modeled reservoirs for each parameter: **(a)** Hydropower and **(b)** in non-hydropower reservoirs.

Fig. 17 depicts the distributions of first-order Sobol indices for each parameter obtained for each of the hydropower and non-hydropower reservoirs for other purpose using a box plot. Overall, Fig. 17 shows that threshold is the most significant parameter. In fact, according to the definition of S_1_, c_threshold_ alone accounts for 48 % of the entire variation in C_2M_ within hydropower reservoirs. This parameter accounts for 74 % of C_2M_ variation in non-hydropower reservoirs. The M parameter is rated second in irrigation reservoirs, accounting for 15 % of all variance in the median, followed by dmax with an S_1_ index of 0.03. The parameter m_start_, which controls the month in which the operating year begins, has very little influence on the total outflow C_2M_ variance, while the effect is significantly greater in non-irrigation reservoirs. For most reservoirs, a and b are regarded as the least relevant characteristics.

### 3.8 The economic impact of water resources management in the rib watershed

Water resource management has a considerable economic influence in the Rib watershed. Water resource management provides for the sustainable use of water in a variety of industries, including agriculture, cattle grazing, forestry, and urban development [16]. Water resource management has the potential to have a large influence on cultivated land in the Rib watershed. Irrigation and drainage systems, for example, can enhance crop yields by ensuring that crops receive the necessary quantity of water. Farmers in the region may benefit from greater agricultural production and profitability as a result of this. Poor water management methods, on the other hand, can have a detrimental influence on cultivated land. Waterlogging and soil salinization, for example, can develop when there is an excess of water in the soil, which can harm crops and diminish yields. Furthermore, a water shortage might limit the quantity of water available for irrigation, reducing crop production. Overall, good water resource management is critical for preserving cultivated land productivity and sustainability in the Rib watershed. Farmers may guarantee that their crops receive the essential quantity of water they need to thrive by employing suitable water management strategies, resulting in enhanced agricultural output and economic growth in the region. Water management for grazing pasture in the Rib watershed has a substantial economic impact. Grazing land is critical to livestock production, which is a significant economic activity in many countries. Effective water resource management in the Rib watershed ensures that water is available for animal drinking and irrigation. This contributes to the health and productivity of grazing land, which in turn promotes cattle growth and development. Proper water management also adds to grazing land sustainability by reducing overgrazing and soil erosion. Water resources may be maintained by limiting water consumption and establishing sustainable grazing methods, resulting in long-term economic advantages for cattle owners and the surrounding community. Furthermore, the Rib watershed's availability of animal water provides a consistent supply of beef, milk, and other livestock products. This helps to ensure food security while also providing economic opportunity for livestock producers and allied sectors. In conclusion, water management is critical to the economic viability of grazing pasture in the Rib watershed. It assures livestock water supply, encourages sustainable land use practices, and supports regional food security and economic prosperity. Effective water resource management, such as limiting water consumption and preventing pollution, can help the Rib watershed maintain and even grow forest cover [17]. Forests have an important role in managing the water cycle, preventing soil erosion, and providing wildlife habitat. Water resource management can assist maintain the long-term viability of the Rib watershed by conserving and sustaining forest cover. Forests in the Rib watershed provide a variety of significant ecological services, including carbon sequestration, water filtration, and wood production. Forests also provide chances for recreation and tourism, which may help local communities produce revenue. As a result, preserving and expanding forest cover via good water resource management can have a favorable economic impact on the Rib watershed. Poor water resource management, on the other hand, can result in deforestation and forest cover deterioration. This can have severe economic consequences, such as soil erosion, biodiversity loss, and a reduction in the supply of lumber and other forest products. Furthermore, damaged forests are less efficient at regulating the water cycle, increasing the danger of floods and droughts. Overall, water resource management is critical to sustaining and improving forest cover in the Rib watershed, which has significant economic ramifications.

### 3.9 Environmental impacts of the reservoir operation and mitigation measures to adverse effects on the downstream ecosystem

It is critical for reservoir operation to control reservoir storage and, hence, reservoir levels with seasonal fluctuations throughout the year [9]. A reservoir's operation can have a variety of environmental effects on the downstream ecosystem, such as changes in water temperature, flow regime, sediment movement, and water quality. These effects can have an influence on aquatic and land species, change ecological processes, and disrupt river-dependent human activities. To mitigate these negative consequences, different mitigation methods such as releasing water to replicate natural flow patterns, controlling water temperature, managing sediment movement, and enhancing water quality through treatment or natural processes such as wetlands can be employed. Dams for reservoirs may have a substantial influence on local populations and their livelihoods. One of the most significant consequences is the relocation of people who live in the region that will be flooded by the reservoir. This can lead to the loss of houses, agriculture, and other resources on which people rely for a living. Dam building can have an influence on the surrounding ecosystem, including changes in water quality, sedimentation patterns, and fish populations, in addition to relocation [11]. These changes may have an influence on the local ecology, possibly affecting other species and the natural resources on which people rely. To prevent these effects, it is critical to include local communities in dam building project planning and decision-making. This might involve ensuring that people are appropriately compensated for any lost land or resources, as well as creating methods to reduce environmental consequences and promote the long-term viability of local livelihoods. Alternative energy sources and conservation techniques should also be considered as viable alternatives to dam development [10].

## 4 Conclusion

In the beginning, the Rib dam was solely intended to be used for irrigation. In this study, it was discovered that additional objectives for the Rib dam might be achieved without hurting the current irrigation needs by employing the irrigation output pipe as a penstock hydropower source. Hydropower can be produced using irrigation water that has been released during the dry season (irrigation period). While during the wet season (when irrigation is not being used), water released through irrigation outlets generates an average of 6.73 MW of hydropower thanks to power plants placed near the dam's toe. Through the controlled outlets, about 209.51 Mm^3^ of water will be released annually for irrigation, environmental purposes, and other downstream water requirements, and the reservoir's average annual intake is 223.54 Mm^3^. In order for Rib Reservoir to produce hydropower, incoming water must be larger than outflow water. According to the present reservoir level, hydrological circumstances, and maximum net volume of water released, this study has established an ideal operation rule or guide curve for the Rib reservoir storing or discharges. This rule or guide curve was developed on an annual average basis. In order to properly manage the reservoir water, guiding curves were created based on the simulation results of the HEC-ResSim model.

## Data availability

Data will be made available on request.

## Funding

No fund has been received from any source to conduct the research work.

## Additional information

No additional information is available for this paper.

## CRediT authorship contribution statement

**Yordanos Mekuriaw Meskr:** Conceptualization, Data curation, Writing - original draft. **Abdella Kemal Mohammed:** Formal analysis, Investigation, Methodology. **Abebe Temesgen Ayalew:** Methodology, Resources, Software. **Tarun Kumar Lohani:** Resources, Software, Supervision, Writing - review & editing.

## Declaration of competing interest

The authors declare that they have no known competing financial interests or personal relationships that could have appeared to influence the work reported in this paper.
